# Sex and Individual Specificity in Behavioral Responses to Chemical Communication and Urinary Volatile Components in Captive Forest Musk Deer (*Moschus berezovskii*)

**DOI:** 10.3390/ani16111610

**Published:** 2026-05-25

**Authors:** Yiting Liu, Qike Wang, Sijia Gao, Kai Li, Dong Zhang

**Affiliations:** 1 School of Ecology and Nature Conservation, Beijing Forestry University, Qinghua East Road 35, Beijing 100083, China; 17613633589@163.com (Y.L.); 17330122439@163.com (S.G.); 2 School of BioSciences, University of Melbourne, Melbourne, VIC 3010, Australia; wang.q@unimelb.edu.au

**Keywords:** forest musk deer, chemical communication, excretions, volatile organic compounds

## Abstract

The forest musk deer (*Moschus berezovskii*) is an endangered species endemic to China. Like many solitary and highly territorial herbivores, forest musk deer rely on chemical signals to convey complex information. However, the chemical composition and functional roles of these signals remain poorly understood. In this study, behavioral assays showed that forest musk deer (8 males and 26 females) exhibited stronger responses to urine than to feces. To characterize the chemical basis of this response, urine samples from five males and five females were analyzed using headspace solid-phase microextraction coupled with gas chromatography–mass spectrometry (HS-SPME-GC-MS), leading to the identification of 83 volatile compounds. Discriminant analyses revealed significant differences in urinary volatile profiles according to both sex and among individuals, with classification accuracies of 100% for sex and 96.7% for individual differentiation. Orthogonal partial least squares discriminant analysis (OPLS-DA) further identified 33 and 17 compounds that potentially convey sex-specific and individual-specific information, respectively, with only nine compounds implicated in both functions. These results suggest that forest musk deer employ a multi-layered coding system in chemical communication, in which distinct chemical compounds encode different types of biological information. Overall, this study provides a framework for understanding chemical signaling in captive forest musk deer and offers insights relevant to the conservation and sustainable management of musk deer populations.

## Simple Summary

The forest musk deer (*Moschus berezovskii*) is an endangered solitary territorial species endemic to China. As with many solitary mammals, chemical communication plays a crucial role in their social interactions. However, the specific functions of urine and feces in conveying information remain poorly understood. In this study, we found that captive forest musk deer exhibited significantly stronger behavioral responses to urine than to feces, indicating that urine may serve as a more important medium for chemical communication. We identified 83 volatile compounds in forest musk deer urine using HS-SPME-GC-MS. Analysis of these volatile profiles revealed that they could reliably distinguish between sexes and even among individuals. Notably, different sets of compounds were responsible for conveying sex-specific and individual-specific information, suggesting a “multi-layered coding” strategy in their chemical communication system. The present study provides the most comprehensive investigation to date on chemical communication in forest musk deer, offering a scientific foundation for improving captive breeding management, reproductive monitoring, and conservation strategies for this endangered species.

## 1. Introduction

Chemical communication plays a vital role in both intraspecific and interspecific interactions among mammals. Mammals use chemical signals and chemical cues to mediate a wide range of social functions, including individual recognition, mate selection, territorial marking, threat defense, status advertisement, and prey localization. In addition, chemical signals can convey information about genetic background, physiological condition, and nutritional status [1,2,3]. The fundamental function of chemical communication is to coordinate potential conflicts of interest among individuals through the transfer of information. This process can maximize survival and reproductive benefits at the group level and profoundly influence the dynamic balance of social structures within populations. Consequently, chemical signals are crucial not only for individual survival and reproduction but also for population stability and the maintenance of genetic diversity [4,5,6,7].

Excretions (feces and urine) represent one of the most important carriers of chemical signals in mammals. Extensive research integrating analyses of volatile compounds in excretions with behavioral responses of receivers has provided substantial insights into chemical communication mechanisms across diverse mammalian species [8,9,10,11]. For example, Rams (*Ovis aries*) can discriminate the estrus status of the opposite sex through urinary odor [12]. In some species, males exhibit a high sensitivity to reproductive chemical cues and may respond to subtle variations in female reproductive status, reflecting a fine-scale level of chemical communication. For instance, exposure to female sex pheromones can induce measurable physiological responses in males, even in the absence of overt behavioral changes, as demonstrated in domestic dogs (*Canis familiaris*) [13]. In red deer (*Cervus canadensis*), males impregnate their abdominal black patches with urine during the rut, producing characteristic odors associated with mate competition [14]. Blackbuck (*Antilope cervicapra*) use urine to signal dominance status [15], and domestic pigs (*Sus scrofa*) utilize urinary chemical cues in social and alarm contexts [16]. Feces also serve as important carriers of chemical information, with volatile compounds and microbial metabolites reliably encoding individual identity, territorial boundaries, and physiological condition. Male Asian elephants (*Elephas maximus*) can detect female reproductive status from fecal cues [17]. Horses (*Equus caballus*) engage in fecal scent marking during the breeding season [18], and goitered gazelles (*Gazella subgutturosa*) combine urine and feces in territorial marking behavior [19]. Because the chemical composition of feces and urine largely reflects individual variation in diet and physiological processes, these excreta can convey reliable information about species identity, sex, individual identity, age, and other biologically relevant traits. Consequently, feces and urine are often considered evolutionarily conserved honest signals in mammalian chemical communication [20].

Although chemical communication has been extensively studied in many terrestrial artiodactyl groups, the chemical communication functions and mechanisms associated with urine and feces in the musk deer family (*Moschidae*) remain largely unexplored. The forest musk deer (*Moschus berezovskii*) is an endangered species endemic to China. Its specialized musk gland secretes musk, a substance of high medicinal and economic value. As a result, the species has experienced prolonged pressure from both overhunting and habitat destruction, leading to severe declines in wild populations and its inclusion on the IUCN Red List [21,22]. Forest musk deer are typically solitary, highly stress-sensitive, and strongly territorial forest-dwelling herbivores. They exhibit centralized defecation behavior, depositing feces repeatedly at fixed locations (latrine sites), and are primarily active during dawn and dusk [23]. Given these ecological and behavioral characteristics, chemical communication is likely to play a central role in intraspecific communication in this species, as observed in other mammals occupying similar ecological niches. However, most previous studies have used feces and urine of forest musk deer primarily as substrates for assessing physiological and pathological conditions. These studies have largely relied on quantitative analyses of hormones and metabolites to monitor parasite infections, immune status, stress levels, and related physiological indicators [24,25]. In contrast, investigations into the potential roles of these excreta in chemical communication, particularly from a behavioral ecology perspective, remain relatively limited, and direct evidence for their communicative functions in forest musk deer is still scarce. To date, existing research has only provided preliminary identification of volatile organic compounds in the urine of male forest musk deer during the musk secretion period, without clarifying their functional roles in chemical communication [26].

Captive forest musk deer represent the main component of the musk deer population in China and largely originate from wild populations across different regions. By 2017, the captive population had exceeded 20,000 individuals and continues to grow while maintaining relatively high genetic diversity [27,28]. Although captive conditions can influence social behavior in ungulates, captive populations generally maintain fundamental physiological processes underlying chemical signal production. Feces and urine are important odor sources in mammalian chemical communication and may convey information related to species identity, sex, individual identity, and physiological condition. Both excreta can also be collected using non-invasive sampling methods. These characteristics make feces and urine promising substrates for investigating the molecular basis and potential functions of chemical communication in forest musk deer.

In this study, we characterized the chemical composition of forest musk deer excretions and examined variation between sexes and among individuals. We also identified candidate compounds potentially involved in chemical communication and evaluated their implications for reproductive regulation and population management. Specifically, we aimed to: (1) compare the roles of feces and urine as media for chemical communication; (2) identify sex-specific and individual-specific patterns in urinary volatile compounds; and (3) determine key chemical signals associated with sex discrimination and individual recognition. These findings provide a scientific basis for understanding chemical communication mechanisms and may contribute to reproductive management in captive populations.

## 2. Materials and Methods

### 2.1. Experimental Animals and Sample Collection

The experimental animals were kept in a captive breeding center in Fengxian, Shaanxi Province, China. All the forest musk deer involved in this study were healthy adults that were fed with the same food and managed in the same procedure.

Feces and urine samples used in the behavioral assays were collected immediately after natural excretion from donor individuals housed in enclosures at a distance from the experimental enclosures. To minimize potential contamination from enclosure substrates, urine samples were collected using a pipette from the surface of the freshly voided urine on the ground, and fecal samples were taken from the inner portion of freshly excreted feces. The experimental subjects had no prior contact with the donors.

For volatile compound analysis of urine, five adult males and five adult females were randomly selected as sample donors (*n* = 5 males and 5 females). Urine samples were collected in September, which falls within the non-breeding season of forest musk deer. At that time, reproductive activity is minimal and the physiological state of the animals is relatively stable. Urine collection followed the same procedure as described for the behavioral experiment for three consecutive days from each individual, resulting in a total of 30 samples. The collected urine was immediately frozen at −20 °C, transported to the laboratory on dry ice, and stored at −80 °C until analysis.

### 2.2. Behavioral Assays

Based on the sex structure, age distribution, and housing management of the population, eight standard enclosures were randomly selected as experimental units (*n* = 8). All individuals housed within these selected enclosures were included in the behavioral observations; no individuals were transferred between enclosures or excluded from their original social groups. In total, the study included the behavior of 34 individuals (8 adult males and 26 adult females, aged 3–7 years). The behavioral assays were conducted during September and October, during which all individuals were maintained under routine feeding and management conditions.

To minimize interference from visual cues, a brick-based odor presentation structure was used. Each structure consisted of three stacked bricks, with two bricks forming the base and one placed on top, creating a concealed compartment to place the samples. Urine samples were absorbed onto perlite and placed in sterile, breathable cloth bags, whereas fecal samples were placed directly into the same bags. All samples were subsequently positioned at the bottom compartment of the odor presentation structure, well below the eye level of the musk deer, ensuring that visual cues were minimized. Prior to the formal experiment, a one-day acclimation was conducted in all enclosures to ensure that the animals adapted to the experimental bricks, exhibited normal behavior, and maintained stable food intake.

The eight enclosures were divided into two groups, each serving as a replicate. Samples within each group were rotated daily, resulting in four replicates. In each enclosure, three concentration gradients and one blank control were established, and the experiment was conducted over four consecutive days. Each treatment was applied only once per day in each enclosure to ensure experimental consistency. Two separate experiments were conducted using fecal and urine samples. For each sample type, treatments included a blank control and three concentration gradients (feces: 2 g, 10 g, and 50 g; urine: 2 mL, 10 mL, and 50 mL). All trials were conducted during the crepuscular activity period of forest musk deer (15:00–18:00) with a 3 h observation period. Sniffing behavior was defined as an individual placing its nose close to the sample device and inhaling continuously for ≥1 s. The total sniffing duration in each enclosure was recorded [29]. Behavioral observations were recorded using video surveillance to avoid disturbing the animals, and behaviors were quantified through video playback analysis.

### 2.3. Urine Sample Processing and HS-SPME Analysis

After natural thawing, 5 mL of urine was transferred to a centrifuge tube and mixed with 1 g NaCl (analytical grade). The mixture was homogenized and centrifuged, and 1 mL of the supernatant was transferred to a 20 mL headspace vial for analysis. A solid-phase microextraction (SPME) fiber (DVB/CAR/PDMS, 10 µm; Supelco, Bellefonte, PA, USA) was preconditioned at 250 °C for 5 min and then exposed to the headspace of the sample vial at 250 °C for 30 min. Desorption was performed in the GC injection port at 250 °C for 5 min, followed by GC–MS analysis [30].

Chemical analyses were conducted using an Agilent 8890-7000E triple quadrupole gas chromatograph coupled with a mass spectrometer (Agilent Technologies, Santa Clara, CA, USA). Volatile compounds were separated on an HP-5ms Ultra Inert capillary column (30 m × 250 μm × 0.25 μm; Agilent Technologies, Santa Clara, CA, USA). The injector operated in splitless mode at 250 °C, with helium (99.999%) as the carrier gas at a constant flow rate of 1.0 mL/min. The oven temperature program was as follows: 45 °C for 2 min; increased to 80 °C at 10 °C/min; increased to 200 °C at 5 °C/min; and finally increased to 280 °C at 20 °C/min and held for 10 min. The total run time was 43.5 min. The transfer line temperature was maintained at 250 °C. Mass spectrometric detection was performed using electron ionization (EI) at 70 eV. The ion source temperature was set at 230 °C and the quadrupole temperature at 150 °C. Data were acquired in full scan mode over a mass range of *m*/*z* 20–450.

Raw GC–MS data were processed using Agilent MassHunter Qualitative Analysis (version 12.0.430). Volatile compounds were tentatively identified by comparison with spectra in the NIST Mass Spectral Library, together with interpretation of fragmentation patterns and retention time comparison. Chromatographic peaks were integrated, and the relative abundance of each compound was calculated using peak area normalization, defined as the peak area of an individual compound divided by the total peak area of all detected compounds within the same sample and expressed as a percentage. To reduce background noise from trace compounds and improve the robustness of multivariate statistical analyses, only compounds with relative abundance greater than 1% were retained for subsequent analyses.

### 2.4. Data Statistical Analysis

To evaluate which odor source can elicit strong behavior response of the forest musk deer, we tested the dose effects of odors on their sniffing behavior, using generalized linear mixed model (GLMM), with the sniffing duration as the response variable, and the odor volume or weight as the fixed effect, and the enclosure identity was treated as a random effect to account for environmental heterogeneity among enclosures.

For the sex difference comparisons, relative abundances of the detected volatile compounds were averaged across repeated samples for each individual, resulting in a single representative chemical profile per individual. Principal component analysis (PCA) was then applied to the averaged dataset to reduce dimensionality and extract the major sources of variation. Subsequently, linear discriminant analysis (LDA) was performed based on the retained principal components to evaluate the ability of urinary volatile compounds to discriminate sex and individual identity. All analyses were conducted using JMP (version 14.0). Orthogonal partial least squares discriminant analysis (OPLS-DA) was conducted using SIMCA (version 14.1), and model robustness was evaluated through permutation testing. Variable importance in projection (VIP) scores were calculated to identify key differential compounds between sexes and among individuals [31].

## 3. Results

### 3.1. Sniffing Behavior Responses to Urine and Feces of Different Concentrations

**Figure 1:**
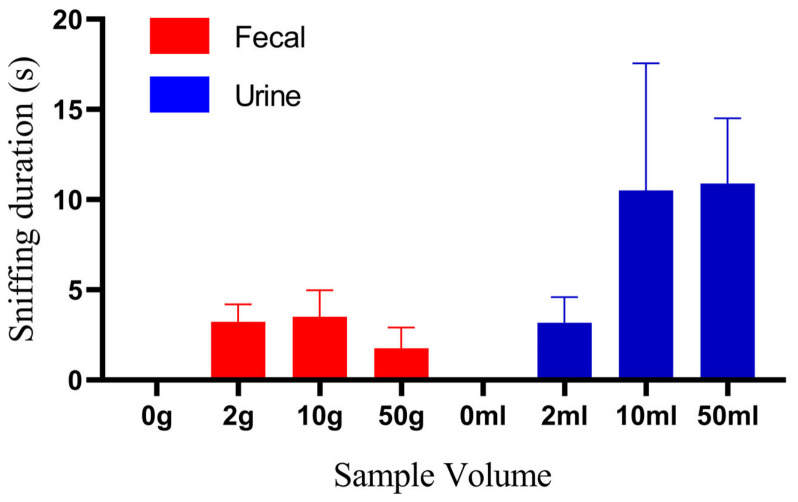
Sniffing duration (seconds) of forest musk deer in response to urine and fecal samples at different concentrations (Mean ± SE).

For urine samples, sniffing duration differed significantly among volume effect (F_3,19.96_ = 5.814, *p* = 0.005), with longer investigation times observed at higher volumes, indicating a clear positive response to increasing odor concentration. In contrast, sniffing duration toward fecal samples did not differ significantly among volume effect (F_3,21_ = 2.763, *p* = 0.067), suggesting a weaker or absent concentration-dependent response (Figure 1). In both urine and feces GLMMs, the random effect of enclosure was not statistically significant (urine: Wald *p* = 0.951; feces: Wald *p* = 0.628), indicating that enclosure identity did not substantially influence sniffing duration.

### 3.2. Volatile Components in Urine

Volatile compounds were analyzed in 30 urine samples collected from captive forest musk deer (Table 1). A total of 83 volatile compounds were identified, each detected in at least two samples, indicating broad compound coverage. Ketones represented the dominant chemical class (36.1%, 30 compounds), including acetophenone and isophorone. Aldehydes and alcohols each accounted for 10.8% (nine compounds each). Phenols, hydrocarbons, nitrogen-containing compounds, and sulfur-containing compounds each represented 7.2% (six compounds per class). Esters accounted for 6.0% (five compounds), while lactones and ethers each represented 3.6% (three compounds each).

**Figure 2:**
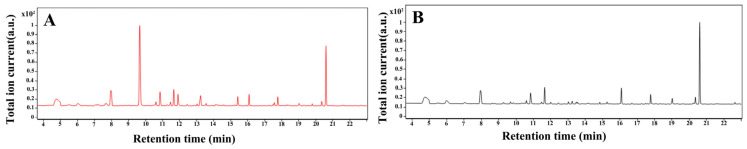
Total ion chromatograms (TIC) of volatile compounds in the urine of different forest musk deer. (**A**) Male. (**B**) Female.

The relative abundance of individual compounds varied substantially among samples (Figure 2). Among these, 2,4-di-tert-butylphenol showed the greatest variation in relative abundance (6.6–52.6%; coefficient of variation = 46%) and the highest mean relative abundance (26%), and was detected in all 30 samples, making it the predominant volatile component in forest musk deer urine. This was followed by 2,3-dihydrobenzofuran (coefficient of variation = 43%) and 4-ethylphenol (coefficient of variation = 42%).

### 3.3. Analysis of Differences Between Sexes and Individuals

**Figure 3:**
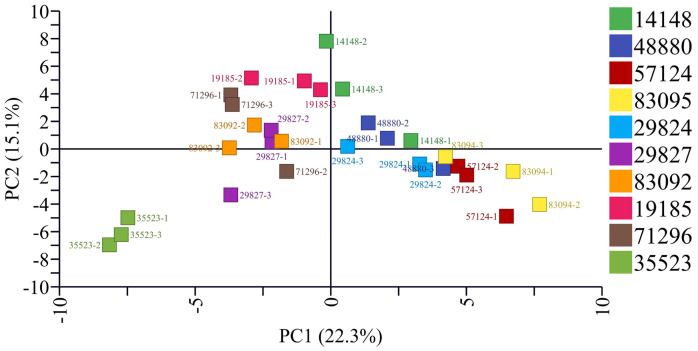
PCA analysis of volatile compounds in urine.

Principal component analysis (PCA) was performed on urinary volatile compound data (Figure 3). The first two principal components (PC1 and PC2) explained 37.4% of the total variance (PC1: 22.3%; PC2: 15.1%). Based on a cumulative variance threshold of 80%, twelve principal components were retained and used as input variables for subsequent discriminant analyses. Linear discriminant analysis revealed strong separation for both sex and individual identity. For sex classification, the eigenvalue was 7.56, explaining 100% of the between-group variance, with a canonical correlation coefficient of 0.939 (Wilks’ Lambda = 0.1167, *p* < 0.001). For individual classification, the eigenvalue was 28.71, explaining 96.7% of the between-group variance, with a canonical correlation coefficient of 0.973 (Wilks’ Lambda = 8.548 × 10^−7^, *p* < 0.001). These results indicate that urinary volatile profiles exhibit strong discriminatory capacity for both sex and individual identity in forest musk deer.

**Figure 4:**
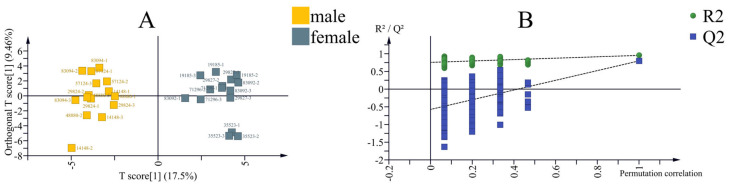
Analysis of urinary volatile compounds in forest musk deer between sexes. (**A**) OPLS-DA score plot for male and female urine samples. (**B**) Permutation test results validating the OPLS-DA model.

Based on the odor characteristics of urinary volatile compounds in male and female forest musk deer, orthogonal partial least squares discriminant analysis (OPLS-DA) was performed using the detected volatile compounds as variables and sex as the grouping factor. The analysis effectively discriminated urine samples by sex and individual. For the sex-specific model, the independent variable fit index (R^2^X) was 0.365, the dependent variable fit index (R^2^Y) was 0.953, and the predictive ability (Q^2^) was 0.797, indicating good model performance. Permutation testing (200 iterations; Figure 4) showed that the Q^2^ regression line intersected the vertical axis below zero, demonstrating the absence of overfitting and confirming model validity. This validation demonstrates that the OPLS-DA model is robust and that urinary volatile profiles provide reliable discrimination between male and female forest musk deer.

**Figure 5:**
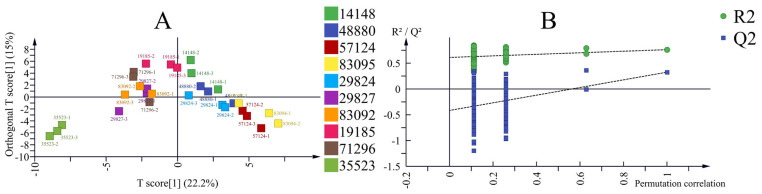
Analysis of urinary volatile compounds in forest musk deer among individuals. (**A**) OPLS-DA score plot distinguishing individual deer. (**B**) Permutation test results validating the OPLS-DA model.

The individual-specific model showed that the independent variable fit index (R^2^X) was 0.709, the dependent variable fit index (R^2^Y) was 0.828, and the cross-validated predictive ability (Q^2^) was 0.457. Permutation testing (200 iterations; Figure 5) indicated that the Q^2^ regression line intersected the vertical axis below zero, demonstrating the absence of overfitting and confirming model validity. These results suggest that urinary volatile compounds in forest musk deer possess a certain capacity for individual discrimination.

**Figure 6:**
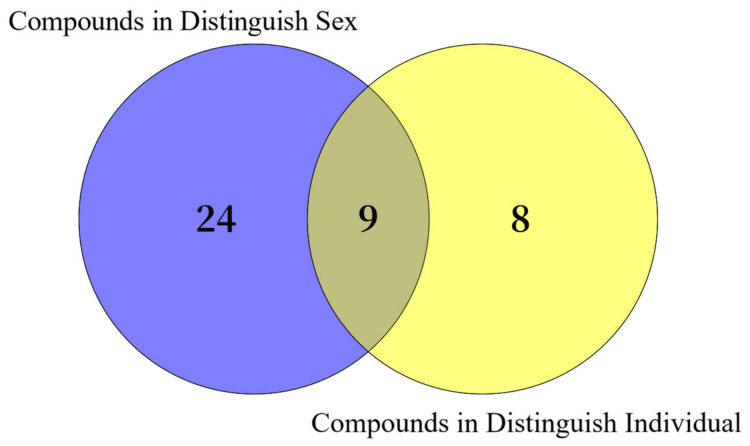
Distribution of urinary volatiles in forest musk deer across sexes and individuals.

To further clarify the contributions of volatile compounds to sex discrimination in forest musk deer chemical communication, compounds meeting the criteria of *p* < 0.05 and VIP > 1 were screened. A total of 33 compounds potentially conveying sex-specific information and 17 compounds potentially conveying individual-specific information were identified. Only nine compounds overlapped between the two groups, suggesting that forest musk deer may use different chemical substances to convey distinct types of information (Figure 6).

## 4. Discussion

This study demonstrates that odor type is a key factor influencing sniffing behavior in forest musk deer, with sniffing duration significantly longer for urine than for feces, indicating a stronger behavioral response to urine. Forest musk deer are crepuscular mammals with peak activity occurring at dawn and dusk, and they exhibit strong territorial behavior. Previous studies have also indicated that this species may be highly sensitive to environmental disturbances and stress. Their solitary lifestyle results in very limited direct encounters between individuals, making efficient and stable chemical communication essential for information exchange. Compared with feces, urine is more closely associated with systemic metabolic processes and hormonal states, thereby providing chemically dynamic cues related to the internal condition of the individual [32,33]. Sniffing duration can be interpreted as an indicator of olfactory interest and attentional engagement toward a stimulus, as behavioral investigation time is widely used in chemical communication studies to reflect the salience and informational value of odor cues [34]. Based on this framework, the longer investigation of urine suggests that urine-derived cues elicit higher behavioral engagement than fecal cues under the tested conditions. In addition, we observed a significant dose-dependent response for urine, whereas feces did not show a comparable pattern. Dose-dependent changes in olfactory exploration have been widely reported in chemical communication systems and are generally interpreted as reflecting behavioral sensitivity to variation in stimulus strength, which may indicate that animals can more accurately detect and respond to graded differences in odor cues [35,36]. Together, these results suggest that urine provides more behaviorally informative olfactory cues under the experimental conditions.

The results of this study suggest that the chemical communication system of forest musk deer may operate through a multi-layered coding strategy, in which different biological information is conveyed through distinct combinations or relative abundances of chemical compounds. Similar mechanisms have been reported in other ungulates. For instance, in white-tailed deer, both sex and social status are encoded by distinct chemical classes of urinary volatiles. Sex-specific differences (male vs. female) involve compound classes such as thiol esters, disulfides, nitriles, and ketals in males, and phenols in females [37]. Social rank differences (dominant vs. subordinate males) are associated with alkanes, alkenes, alcohols, and a nitrile in dominants, whereas subordinates exhibit a broader range of compounds, including thiol esters, disulfides, amines, and benzenes [38]. In red deer, reproductive state (rutting males vs. non-rutting individuals) is characterized by an increase in medium-chain carboxylic acids and their derivatives (including amides and lactones), while age-related differences (calves vs. adults) are associated with a distinct set of unidentified compounds [39]. In water buffalo, different reproductive stages (diestrus and pregnancy) and sex are each associated with unique compound classes, including alkanes, iodo-compounds, phthalates, and alcohols, with no single compound being exclusive to estrus [40]. Together, these examples demonstrate that sex, social rank, reproductive state, age, and pregnancy are each associated with qualitatively distinct chemical classes of urinary volatiles, supporting a multi-layered coding strategy in ungulate chemical communication. Similar combinatorial coding has also been documented in mice (major urinary proteins), lizards (femoral gland secretions), insects, and plants, where different compound combinations convey multiple types of information [41,42,43].

The volatile compounds detected in forest musk deer urine mainly consisted of alcohols, ketones, aldehydes, and sulfur-containing compounds. These compounds may originate from three major sources: plant-derived secondary metabolites, endogenous metabolic products, and microbial transformation products. Similar to other mammals, urinary volatiles reflect the combined effects of genetic background, physiological metabolism, diet, and microbial activity [44,45,46]. Plant-derived metabolites may indicate dietary preferences and habitat characteristics, while endogenous metabolites are influenced by hormonal regulation and genetic factors, enabling relatively stable encoding of sex and individual identity [47,48]. Microbial metabolism within the gastrointestinal and urinary systems can further modify these compounds, generating additional volatile substances through fermentation and degradation processes [49]. Together, these sources form a complex chemical signal library capable of conveying multiple types of biological information.

Compared with other reported ungulate species, the volatile compounds identified in forest musk deer urine exhibit both common features and species-specific characteristics. For instance, alcohols, aldehydes, and ketones similar to those detected in the present study have previously been reported in the urine and vaginal mucus of estrous female white-tailed deer (*Odocoileus virginianus*) [37]. Aromatic compounds have also been identified in the abdominal black-spot secretions of red deer [14]. Notably, phenolic compounds (e.g., 2,4-di-tert-butylphenol and 4-ethylphenol) and pyrazine derivatives have been repeatedly reported in chemical communication studies across multiple species, suggesting that these compound classes may play important roles in odor-mediated signaling. Among the compounds detected here, 2,4-di-tert-butylphenol exhibited both the highest detection frequency and the highest relative abundance. This compound also shows similarity to antioxidant-related compounds previously identified in red deer scent secretions [14], suggesting that it may participate in analogous information-transfer processes in intraspecific chemical communication among ungulates. Furthermore, sulfur-containing compounds and ketones identified in the present study have also been associated with sex recognition or reproductive status in species such as the maned wolf and horse [18,50]. The occurrence of similar chemical constituents across multiple mammalian species further supports the possibility that certain classes of volatile compounds constitute a conserved component of mammalian chemical signaling systems [51].

Understanding chemical communication in forest musk deer is also important for improving captive breeding management. Captive populations often exhibit slow growth rates and unstable reproductive success. Identifying chemical markers associated with reproductive status and individual identity could provide valuable tools for non-invasive monitoring. Volatile organic compounds (VOCs) have recently shown strong potential as biological indicators in animal behavioral and physiological studies [52]. The present study characterized urinary VOC profiles in captive forest musk deer during the non-breeding season and identified significant sex- and individual-level differences. These findings provide a foundation for developing odor-based techniques for estrus detection, health monitoring, and individual identification.

Currently, captive breeding programs typically rely on natural mating through temporary co-housing of males and females. However, the species’ high sensitivity to stress often results in aggressive interactions and missed mating opportunities. The sex-specific urinary compounds identified in this study may provide chemical cues involved in sex recognition, offering a potential basis for developing olfactory-informed pairing strategies. While these findings may help guide future efforts to improve mating management in captive populations, their direct role in reproductive compatibility remains to be further investigated.

Future studies should further investigate seasonal variation in urinary volatile compounds, particularly during the breeding season, and evaluate the behavioral responses of conspecifics to candidate signal compounds through controlled bioassays. Integrating chemical, behavioral, and physiological approaches will help clarify the functional roles of these compounds and deepen our understanding of chemical communication mechanisms in forest musk deer.

## 5. Conclusions

This study indicates that urine-derived volatile compounds elicit stronger sniffing responses than fecal volatiles in forest musk deer. A total of 83 volatile compounds were identified in urine. Urinary volatile profiles effectively distinguished sex and individual identity, with 33 compounds associated with sex-specific information and 17 with individual-specific information; only nine compounds were shared between the two categories. These findings support a multi-layered coding strategy in the chemical communication system of forest musk deer. The identified compounds provide a scientific basis for developing non-invasive approaches for estrus detection, individual identification, and mate compatibility assessment, thereby contributing to the conservation and sustainable management of this endangered species.

## Figures and Tables

**Table 1:** Volatile organic compound components in the urine of forest musk deer.

No.	RT (min)	Compound	Molecular Formula	Molecular Weight	CAS No.	Sex	Individual
1	5.491	p-Xylene	C_8_H_10_	106	106-42-3	Y	Y
2	5.997	Oxime-, methoxy-phenyl-_	C_8_H_9_NO_2_	151	67160-14-9	N	Y
3	7.182	Benzaldehyde	C_7_H_6_O	106	100-52-7	N	N
4	7.421	2-Hepten-4-one, 6-methyl-	C_8_H_14_O	126	49852-35-9	N	N
5	7.544	Phenol	C_6_H_6_O	94	108-95-2	N	N
6	7.633	n-Caproic acid vinyl ester	C_8_H_14_O_2_	142	3050-69-9	Y	N
7	7.687	2,3-Octanedione	C_8_H_14_O_2_	142	585-25-1	N	N
8	8.486	4-Methylidenecyclohexan-1-one	C_7_H_10_O	110	29648-66-6	Y	N
9	8.602	1-Hexanol, 2-ethyl-	C_8_H_18_O	130	104-76-7	Y	N
10	8.749	Benzyl alcohol	C_7_H_8_O	108	100-51-6	N	N
11	8.871	Cycloheptanol, 2-methylene	C_8_H_14_O	126	16240-38-3	Y	N
12	9.003	5-Ethylcyclopent-1-enecarboxaldehyde	C_8_H_12_O	124	36431-60-4	N	Y
13	9.143	Cyclohexanone, 2-(1-methylethylidene)-	C_9_H_14_O	138	13747-73-4	N	N
14	9.28	Octane, 3,3-dimethyl-	C_10_H_22_	142	4110-44-5	N	N
15	9.372	3,6,6-Trimethyl-Cyclohex-2-Enone	C_9_H_14_O	138	23438-77-9	Y	Y
16	9.535	Acetophenone	C_8_H_8_O	120	98-86-2	Y	N
17	9.846	Benzofuran, 2,3-dihydro-	C_8_H_8_O	120	496-16-2	N	N
18	9.974	Benzene, 1-ethyl-3,5-dimethyl-	C_10_H_14_	134	934-74-7	N	N
19	10.169	6-Nonenal, 3,7-dimethyl-	C_11_H_20_O	168	36557-62-7	Y	N
20	10.249	2-Ethyl-3-methoxy-2-cyclopentenone	C_8_H_12_O_2_	140	25112-86-1	Y	Y
21	10.34	Undecane	C_11_H_24_	156	1120-21-4	N	N
22	10.428	6-Methyl-3,5-heptadiene-2-one	C_8_H_12_O	124	1604-28-0	N	N
23	10.75	N-Benzylideneethylamine	C_9_H_11_N	133	6852-54-6	N	N
24	10.839	Isophorone	C_9_H_14_O	138	78-59-1	N	N
25	11.033	Benzyl methyl ketone	C_9_H_10_O	134	103-79-7	N	N
26	11.29	3-Nonen-2-one	C_9_H_16_O	140	14309-57-0	N	N
27	11.41	4,4-Dimethyl-2-propenylcyclopentanone	C_10_H_16_O	152	68261-88-1	N	N
28	11.473	2-Isopropenyl-2,5-dimethyl-1-cyclohexanone	C_11_H_18_O	166	6711-26-8	Y	Y
29	11.733	Sabinone	C_10_H_14_O	150	67690-48-6	N	N
30	11.904	Phenol, 4-ethyl-	C_8_H_10_O	122	123-07-9	N	N
31	12.000	1,4-Cyclohexanedione, 2,2,6-trimethyl-	C_9_H_14_O_2_	154	20547-99-3	N	N
32	12.212	Benzenamine, 2,3-dimethoxy-	C_8_H_11_NO_2_	153	6299-67-8	Y	N
33	12.439	Naphthalene	C_10_H_8_	128	91-20-3	Y	N
34	12.509	4,4-Dimethyl-2-allylcyclohexanone	C_11_H_18_O	166	59077-96-2	Y	N
35	12.717	Methyl salicylate	C_8_H_8_O_3_	152	119-36-8	N	N
36	12.854	1,3-Cyclohexadiene-1-carboxaldehyde, 2,6,6-trimethyl-	C_10_H_14_O	150	116-26-7	N	Y
37	12.956	5-Isopropenyl-1,2-dimethylcyclohex-2-enol	C_11_H_18_O	166	85710-64-1	N	N
38	13.025	Cyclohexane, [(1-methylpropyl)thio]-	C_10_H_20_S	172	7133-22-4	N	N
39	13.181	2-Nonen-4-one, 2-methyl-	C_10_H_18_O	154	2903-23-3	Y	Y
40	13.24	Benzaldehyde, 2,5-dimethyl-	C_9_H_10_O	134	5779-94-2	N	N
41	13.387	1-Cyclohexene-1-carboxaldehyde, 2,6,6-trimethyl-	C_10_H_16_O	152	432-25-7	Y	N
42	13.476	Benzothiazole	C_7_H_5_NS	135	95-16-9	N	N
43	13.849	Quinoline	C_9_H_7_N	129	91-22-5	N	N
44	14.337	Cyclopropanecarboxaldehyde, 2-methyl-2-(4-methyl-3-pentenyl)-	C_11_H_18_O	166	97231-35-1	N	N
45	14.453	alpha-Ionone	C_13_H_20_O	192	127-41-3	Y	N
46	14.585	1,4,4,7a-Tetramethyl-2,4,5,6,7,7a-hexahydro-1H-indene-1,7-diol	C_13_H_22_O_2_	210	121747-53-3	Y	N
47	14.699	5,8-Decadien-2-one, 5,9-dimethyl-, (E)-	C_12_H_20_O	180	130876-99-2	Y	N
48	14.835	Dodecane, 2,6,11-trimethyl-	C_15_H_32_	212	31295-56-4	N	N
49	15.247	Benzothiazole, 2-methyl-	C_8_H_7_NS	149	120-75-2	Y	N
50	15.417	Ethanone, 1-(2-aminophenyl)-	C_8_H_9_NO	135	551-93-9	N	Y
51	15.804	(1,2,4-Trimethylcyclohexyl)methanol	C_10_H_20_O	156	1217733-74-8	Y	N
52	15.929	3-Buten-2-one, 4-(2-hydroxy-2,6,6-trimethylcyclohexyl)-	C_13_H_22_O_2_	210	55955-46-9	N	N
53	16.183	Cyclopropanemethanol, 2-methyl-2-(4-methyl-3-pentenyl)-	C_11_H_20_O	168	98678-70-7	Y	N
54	16.247	(2,2,6-Trimethyl-bicyclo[4.1.0]hept-1-yl)-methanol	C_11_H_20_O	168	78996-11-9	Y	N
55	16.458	4-Oxo-2-(E)-nonenal	C_9_H_14_O_2_	154	103560-62-9	N	N
56	16.683	Phenol, 2-(1,1-dimethylethyl)-5-methyl-	C_11_H_16_O	164	88-60-8	Y	Y
57	16.722	1-(1-Hydroxy-1-methylethyl)-4-methylbicyclo[3.1.0]hexan-3-one	C_10_H_16_O_2_	168	183296-46-0	Y	Y
58	16.965	2(3H)-Furanone, dihydro-5-pentyl-	C_9_H_16_O_2_	156	104-61-0	N	N
59	17.207	Cyclohexanol, 1-(1-hexenyl)-, (E)-	C_12_H_22_O	182	34678-40-5	N	N
60	17.257	Propanoic acid, 2-methyl-, 3-hydroxy-2,2,4-trimethylpentyl ester	C_12_H_24_O_3_	216	77-68-9	N	N
61	17.556	2-Buten-1-one, 1-(2,6,6-trimethyl-1,3-cyclohexadien-1-yl)-, (E)-	C_13_H_18_O	190	23726-93-4	Y	N
62	17.672	2-Buten-1-one, 1-(2,6,6-trimethyl-1-cyclohexen-1-yl)-	C_13_H_20_O	192	35044-68-9	Y	N
63	17.771	Phenol, 4-(1,1-dimethylpropyl)-	C_11_H_16_O	164	80-46-6	N	N
64	17.956	Diphenyl ether	C_12_H_10_O	170	101-84-8	N	N
65	18.189	10-Undecenoic acid, methyl ester	C_12_H_22_O_2_	198	111-81-9	N	N
66	18.302	Damascone, β-	C_13_H_20_O	192	23726-91-2	Y	N
67	18.406	2-Cyclopenten-1-one, 2-methyl-3-pentyl-	C_11_H_18_O	166	5739-17-3	Y	N
68	18.785	2,5,6-Trimethyl-1,3-benzothiazole	C_10_H_11_NS	177	5683-41-0	Y	N
69	19.016	Quinoline, 1,2-dihydro-2,2,4-trimethyl-	C_12_H_15_N	173	147-47-7	N	Y
70	19.07	Furan, 2-methyl-5-(1,1,5-trimethyl-5-hexenyl)-	C_14_H_22_O	206	77143-15-8	N	Y
71	19.308	3-Buten-2-one, 4-(4-hydroxy-2,6,6-trimethyl-1-cyclohexen-1-yl)-, (3E)-	C_13_H_20_O_2_	208	116296-75-4	Y	N
72	19.586	2(3H)-Furanone, 5-hexyldihydro-	C_10_H_18_O_2_	170	706-14-9	N	N
73	19.797	Jasmone lactone, 3Z-	C_11_H_18_O_2_	182	70851-61-5	Y	Y
74	20.045	β-Ionone	C_13_H_20_O	192	79-77-6	N	N
75	20.153	2-Butenal, 2-methyl-4-(2,6,6-trimethyl-1-cyclohexen-1-yl)-	C_14_H_22_O	206	3155-71-3	N	N
76	20.224	2-Cyclohexen-1-one, 4-(3-hydroxybutyl)-3,5,5-trimethyl-	C_13_H_22_O_2_	210	36151-02-7	N	N
77	20.599	2,4-Di-tert-butylphenol	C_14_H_22_O	206	96-76-4	N	Y
78	20.717	Menadione	C_11_H_8_O_2_	172	58-27-5	N	N
79	20.829	2-Cyclohexen-1-one, 4-hydroxy-4-(1-methylethyl)-	C_9_H_14_O_2_	154	39725-34-3	Y	Y
80	20.97	Benzothiazole, 2-butyl-	C_11_H_13_NS	191	54798-95-7	Y	N
81	21.091	1,3-Benzenediol, 5-pentyl-	C_11_H_16_O_2_	180	500-66-3	N	N
82	22.634	2,2,4-Trimethyl-1,3-pentanediol diisobutyrate	C_16_H_30_O_4_	286	6846-50-0	N	Y
83	22.695	2(3H)-Benzothiazolethione, 3-methyl	C_8_H_7_NS_2_	181	2254-94-6	N	N

Note: The “Sex” and “Individual” columns indicate whether a compound contributed significantly to discrimination between sexes or among individuals, respectively. “Y” denotes a significant contribution, while “N” indicates no significant contribution.

## Data Availability

Data are available from the corresponding authors upon reasonable request.

## References

[1] Caro S.P., Balthazart J., Bonadonna F. (2015). The Perfume of Reproduction in Birds: Chemosignaling in Avian Social Life. Horm. Behav..

[2] Johansson B.G., Jones T.M. (2007). The Role of Chemical Communication in Mate Choice. Biol. Rev..

[3] Liu D.Z., Tian H. (2010). Functions and Underlying Mechanisms of Chemical Communication in Animals. Chin. J. Nat..

[4] Brennan P.A., Zufall F. (2006). Pheromonal Communication in Vertebrates. Nature.

[5] Campbell-Palmer R., Rosell F. (2011). The Importance of Chemical Communication Studies to Mammalian Conservation Biology: A Review. Biol. Conserv..

[6] Marin A.C., Schaefer A.T., Ackels T. (2021). Spatial Information from the Odour Environment in Mammalian Olfaction. Cell Tissue Res..

[7] Slade B.E., Schulte B.A., Rasmussen L.E.L. (2003). Oestrous State Dynamics in Chemical Communication by Captive Female Asian Elephants. Anim. Behav..

[8] Lai S.-C., Vasilieva N.Y., Johnston R.E. (1996). Odors Providing Sexual Information in Djungarian Hamsters: Evidence for an Across-Odor Code. Horm. Behav..

[9] Krueger K., Flauger B. (2011). Olfactory Recognition of Individual Competitors by Means of Faeces in Horse (*Equus caballus*). Anim. Cogn..

[10] Rossini C., Ungerfeld R. (2016). Chemical Profile of the Cutaneous Gland Secretions from Male Pampas Deer (*Ozotoceros bezoarticus*). J. Mammal..

[11] McLean S., Nichols D.S., Davies N.W. (2021). Volatile Scent Chemicals in the Urine of the Red Fox, *Vulpes vulpes*. PLoS ONE.

[12] Blissitt M.J., Bland K.P., Cottrell D.F. (1990). Olfactory and Vomeronasal Chemoreception and the Discrimination of Oestrous and Non-Oestrous Ewe Urine Odours by the Ram. Appl. Anim. Behav. Sci..

[13] Dzięcioł M., Stańczyk E., Noszczyk-Nowak A., Niżański W., Ochota M., Kozdrowski R. (2012). Influence of Bitches Sex Pheromones on the Heart Rate and Other Chosen Parameters of Blood Flow in Stud Dogs (*Canis familiaris*). Res. Vet. Sci..

[14] Martín J., Carranza J., López P., Alarcos S., Pérez-González J. (2014). A New Sexual Signal in Rutting Male Red Deer: Age Related Chemical Scent Constituents in the Belly Black Spot. Mamm. Biol..

[15] Rajagopal T., Archunan G., Geraldine P., Balasundaram C. (2010). Assessment of Dominance Hierarchy through Urine Scent Marking and Its Chemical Constituents in Male Blackbuck Antelope Cervicapra, a Critically Endangered Species. Behav. Process..

[16] Sankarganesh D., Kirkwood R., Angayarkanni J., Achiraman S., Archunan G. (2021). Pig Pheromones and Behaviors: A Review. Theriogenology.

[17] Ghosal R., Seshagiri P.B., Sukumar R. (2012). Dung as a Potential Medium for Inter-Sexual Chemical Signaling in Asian Elephants (*Elephas maximus*). Behav. Process..

[18] Kimura R. (2001). Volatile Substances in Feces, Urine and Urine-Marked Feces of Feral Horses. Can. J. Anim. Sci..

[19] Blank D., Ruckstuhl K., Yang W. (2015). The Economics of Scent Marking with Urine and Feces in Goitered Gazelle (*Gazella subgutturosa*). Mamm. Res..

[20] Zala S.M. (2004). Scent-Marking Displays Provide Honest Signals of Health and Infection. Behav. Ecol..

[21] Yang Q., Meng X., Xia L., Feng Z. (2003). Conservation Status and Causes of Decline of Musk Deer (*Moschus* spp.) in China. Biol. Conserv..

[22] Bo X., Chen J., Mu J., Dong X., Ren Z., Liu J., Wang S. (2024). Quercetin Promotes the Secretion of Musk by Regulating the Hormone Level and Microbial Structure of Forest Musk Deer. Integr. Zool..

[23] Singh P.B., Saud P., Jiang Z., Zhou Z., Hu Y., Hu H. (2022). Himalayan Musk Deer (*Moshcus leucogaster*) Behavior at Latrine Sites and Their Implications in Conservation. Ecol. Evol..

[24] Gao Y., Duszynski D.W., Yuan F., Hu D., Zhang D. (2021). Coccidian Parasites in the Endangered Forest Musk Deer (*Moschus berezovskii*) in China, with the Description of Six New Species of Eimeria (Apicomplexa: Eimeriidae). Parasite.

[25] Zhou X., Lv Q., Qin Y., Yuan N., Li Y., Zhou M., Meng X. (2023). Effects of Social Stress on the Welfare of Captive Male Alpine Musk Deer: Stereotypic Behavior, Fecal Cortisol, and Musk Secretion. Appl. Anim. Behav. Sci..

[26] Xian Y.K., Jiang J.Q., Wang C.X., Dai X.Y., Wang J.M., Wu J., Li Y.D., Cai Y.H. (2019). Preliminary Analysis of Volatile Compounds in Urine of Male Forest Musk Deer in the Early and Later Period of Musk Secretion. J. Econ. Anim..

[27] Jiang F., Zhang J., Song P., Xu B., Cai Z., Li X., Gao H., Gu H., Zhang T. (2026). Conservation Status, Decline Factors, and Strategies for Globally Endangered Musk Deer (*Moschus* spp.) in China. Wildl. Lett..

[28] Wang Z., Lu G., Gao Y., Yan L., Li M., Hu D., Zhang D. (2023). mtDNA CR Evidence Indicates High Genetic Diversity of Captive Forest Musk Deer in Shaanxi Province, China. Animals.

[29] Papes F., Nakahara T.S., Camargo A.P., Simoes De Souza F.M., Antunes G. (2018). Behavioral Assays in the Study of Olfaction: A Practical Guide. Olfactory Receptors.

[30] Živković Semren T., Brčić Karačonji I., Safner T., Brajenović N., Tariba Lovaković B., Pizent A. (2018). Gas Chromatographic-Mass Spectrometric Analysis of Urinary Volatile Organic Metabolites: Optimization of the HS-SPME Procedure and Sample Storage Conditions. Talanta.

[31] Yu M., Luobu Z., Zhuoga D., Wei X., Tang Y. (2025). Physiological and Broadly Targeted Metabolomic Analyses of Barley (*Hordeum vulgare* L.) in Response to Low-Temperature Stress. BMC Genom..

[32] Fritz W.F., Becker S.E., Katz L.S. (2021). Urine from Domesticated Male Goats (*Capra hircus*) Provides Attractive Olfactory Cues to Estrous Females. Appl. Anim. Behav. Sci..

[33] Fujita S., Mitsunaga F., Sugiura H., Shimizu K. (2001). Measurement of Urinary and Fecal Steroid Metabolites during the Ovarian Cycle in Captive and Wild Japanese Macaques, *Macaca fuscata*. Am. J. Primatol..

[34] Shirasu M., Ito S., Itoigawa A., Hayakawa T., Kinoshita K., Munechika I., Imai H., Touhara K. (2020). Key Male Glandular Odorants Attracting Female Ring-Tailed Lemurs. Curr. Biol..

[35] Manoel D., Makhlouf M., Arayata C.J., Sathappan A., Da’as S., Abdelrahman D., Selvaraj S., Hasnah R., Mainland J.D., Gerkin R.C. (2021). Deconstructing the Mouse Olfactory Percept through an Ethological Atlas. Curr. Biol..

[36] Rasmussen L.E.L. (1999). Evolution of Chemical Signals in the Asian Elephant, Elephas maximus: Behavioural and Ecological Influences. J. Biosci..

[37] Jemiolo B., Miller K.V., Wiesler D., Jelinek I., Novotny M., Marchinton R.L. (1995). Putative Chemical Signals from White-Tailed Deer (*Odocoileus virginianus*): Urinary and Vaginal Mucus Volatiles Excreted by Females during Breeding Season. J. Chem. Ecol..

[38] Miller K.V., Jemiolo B., Gassett J.W., Jelinek I., Wiesler D., Novotny M. (1998). Putative Chemical Signals from White-Tailed Deer (Odocoileus virginianus): Social and Seasonal Effects on Urinary Volatile Excretion in Males. J. Chem. Ecol..

[39] Bakke J.M., Figenschou E. (1990). Volatile Compounds from the Red Deer (*Cervus elaphus*): Substances Secreted via the Urine. Comp. Biochem. Physiol. A Physiol..

[40] Barman P., Yadav M.C., Kumar H., Meur S.K., Ghosh S.K. (2013). Gas Chromatographic-Mass Spectrometric Analysis of Chemical Volatiles in Buffalo (*Bubalus bubalis*) Urine. Theriogenology.

[41] Kaur A.W., Ackels T., Kuo T.-H., Cichy A., Dey S., Hays C., Kateri M., Logan D.W., Marton T.F., Spehr M. (2014). Murine Pheromone Proteins Constitute a Context-Dependent Combinatorial Code Governing Multiple Social Behaviors. Cell.

[42] Martín J., López P. (2015). Condition-Dependent Chemosignals in Reproductive Behavior of Lizards. Horm. Behav..

[43] Junker R.R., Kuppler J., Amo L., Blande J.D., Borges R.M., Van Dam N.M., Dicke M., Dötterl S., Ehlers B.K., Etl F. (2018). Covariation and Phenotypic Integration in Chemical Communication Displays: Biosynthetic Constraints and Eco-evolutionary Implications. New Phytol..

[44] Wood W.F., Copeland J.P., Yates R.E., Horsey I.K., McGreevy L.R. (2009). Potential Semiochemicals in Urine from Free Ranging Wolverines (*Gulo gulo Pallas*, 1780). Biochem. Syst. Ecol..

[45] Ma W., Klemm W.R. (1997). Variations of Equine Urinary Volatile Compounds during the Oestrous Cycle. Vet. Res. Commun..

[46] Wang W., He L., Liu B., Li L., Wei N., Zhou R., Qi L., Liu S., Hu D. (2015). Feeding Performance and Preferences of Captive Forest Musk Deer While on a Cafeteria Diet. Folia Zool..

[47] Chandran A.K., Stach M., Kucharska A.Z., Sokół-Łętowska A., Szumny A., Moreira H., Szyjka A., Barg E., Kolniak-Ostek J. (2025). Comparison of Polyphenol and Volatile Compounds and in Vitro Antioxidant, Anti-Inflammatory, Antidiabetic, Anti-Ageing, and Anticancer Activities of Dry Tea Leaves. LWT.

[48] Zhou Y., Rui L. (2010). Major Urinary Protein Regulation of Chemical Communication and Nutrient Metabolism. Vitamins & Hormones.

[49] Goodwin T.E., Broederdorf L.J., Burkert B.A., Hirwa I.H., Mark D.B., Waldrip Z.J., Kopper R.A., Sutherland M.V., Freeman E.W., Hollister-Smith J.A. (2012). Chemical Signals of Elephant Musth: Temporal Aspects of Microbially-Mediated Modifications. J. Chem. Ecol..

[50] Jones M.K., Huff T.B., Freeman E.W., Songsasen N. (2021). Differential Expression of Urinary Volatile Organic Compounds by Sex, Male Reproductive Status, and Pairing Status in the Maned Wolf (*Chrysocyon brachyurus*). PLoS ONE.

[51] Root-Gutteridge H., De Kock N., Young M., Gill A.C., Penny J.A., Pike T.W., Mills D.S. (2025). Common Scents? A Review of Potentially Shared Chemical Signals in the Order Carnivora. Chem. Senses.

[52] Moura P.C., Raposo M., Vassilenko V. (2023). Breath Volatile Organic Compounds (VOCs) as Biomarkers for the Diagnosis of Pathological Conditions: A Review. Biomed. J..

